# Risk factors for mortality in periprosthetic femur fractures about the hip-a retrospective analysis

**DOI:** 10.1007/s00264-024-06346-7

**Published:** 2024-10-10

**Authors:** Katharina Müller, Samira Zeynalova, Johannes K.M. Fakler, Christian Kleber, Andreas Roth, Georg Osterhoff

**Affiliations:** 1 Department of Orthopaedic, Trauma and Plastic Surgery, University Hospital of Leipzig, Liebigstr. 20, 04103 Leipzig, Germany; 2 Institute for Medical Informatics, Statistics and Epidemiology (IMISE), University of Leipzig, Härtelstrasse 16-18, 04107 Leipzig, Germany; 3 Department of Orthopaedic and Trauma Surgery, Hospital of Passau, Innstr. 76, 94032 Passau, Germany

**Keywords:** Periprosthetic fracture, Total hip arthroplasty, Vancouver classification, Hip fracture, Mortality, Time to surgery, Time of surgery

## Abstract

**Purpose:**

Fractures around the hip are known to be an indicator for fragility and are associated with high mortality and various complications. A special type of fractures around the hip are periprosthetic femur fractures (PPF) after Total Hip Arthroplasty (THA). The aim of this study was to investigate the mortality rate associated with PPF after THA and to identify risk factors that may increase it.

**Methods:**

Consecutive patients (*N* = 158) who were treated for a PPF after THA in our university hospital between 2010 and 2020 were identified and mortality was assessed using the residential registry. Univariate (Kaplan-Meier-Estimator) and multivariate (Cox-Regression) statistical analysis was performed to identify risk factors influencing mortality.

**Results:**

One-year-mortality rate was 23.4% and 2-year mortality was 29.2%. Mortality was significantly influenced by age, gender, treatment, type of comorbidity and time of surgery (*p* < 0.05). Surgical treatment during regular working hours (8 to 18 h) reduced mortality by 53.2% compared to surgery on call (OR: 0.468, 95% CI 0.223, 0.986; *p* = 0.046). For every year of age, mortality risk increased by 12.9% (OR: 1,129, 95% CI 1.078, 1.182; *p* < 0.001). The type of fracture according to the Vancouver classification had no influence on mortality (*p* = 0.179). Plate fixation and conservative treatment were associated with a higher mortality compared to revision arthroplasty (plate: OR 2.8, 95% CI 1.318, 5.998; *p* = 0.007; conservative: OR 2.5, 95% CI 1.421, 4.507; *p* = 0.002).

**Conclusion:**

Surgical treatment during regular working hours is associated with lower mortality compared to surgery outside these hours. In this retrospective cohort, time to surgery showed no significant impact on all-cause mortality, and revision arthroplasty was associated with lower mortality than conservative treatment or plate fixation.

**Level of evidence:**

IV (Retrospective cohort study).

## Introduction

Periprosthetic fractures of the femur (PPF) are challenging injuries in patients with previous total hip arthroplasty (THA) or total knee arthroplasty (TKA). They can occur early as a complication of the initial intervention itself or later as a consequence of aseptic loosening or trauma or both [16, 18].

The German Endoprosthesis Register (EPRD) reports 177,826 primary THA implants for 2022. The report suggests that 15.9% of revision surgeries following first time THA were being caused by PPF. Patients with PPF after THA showed a certain risk profile: median age of 81 years, female sex, average BMI of 25.8 km/m^3^ and ASA score of 2.7 [8]. Other studies also identified female sex, age above 65 years and a BMI under 25 km/m^3^ as risk factors for PPF [15]. Uncemented prosthesis seem to be more inclined for intra-operative as well as post-operative PPF [1, 11].

As with other fractures, the goals of treatment include fracture union, restoration of alignment, and return to the preinjury level of function without pain. In addition, treatment aims to maintain the stability of the prosthesis and - if needed - to restore an adequate bone stock. If the femoral stem has good anchorage, PPF around the hip can usually be treated by open reduction internal fixation (ORIF). In fractures where the femoral stem is loose revision THA or long femoral stems are used and the fracture fragment will be fixed to the stem by a plate or cerclage wires if necessary. Guidelines for treatment of PPF are usually based on the Vancouver classification [5, 22].

In the literature, the management of PPF is often compared to hip fractures without a preexisting implant as they have a similar patient collective, mortality rates and injury mechanism [3, 7]. It is generally acknowledged to treat native hip fractures within a maximum timeframe of 24 to 48 h until surgery as this showed improved survival [2, 17]. Some studies suggest that surgery within 24 h after hospital admittance is also beneficial for mortality and morbidity in PPF [4, 7]. Fractures around the hip are known to be an indicator for fragility and are associated with high mortality [3, 12, 13, 21]. A recent meta-analysis reported an inpatient mortality of PPF with 2.4% and a one year mortality of 13.4% [12].

As PPF will affect more and more patients, it is of interest to observe how these patient-related risk factors as well as different treatments influence the outcome after PPF. The high mortality rates show the need to further optimize hospital care and treatment for this patient collective.

The aim of this study was to investigate the mortality rate associated with PPF after THA and to identify risk factors that may increase it.

### Patients and methods

This retrospective cohort study was approved by the institutional ethics committee (409/20-ek) .

Patients aged older than 18 years who were treated for a PPF around the hip and were admitted between 2009 and 2020 to the University Hospital of Leipzig were identified.

Charts were reviewed for patient related factors included age, gender, BMI, type and standing time of primary THA, comorbidities (cardiovascular disease, pulmonary disease, dementia, other neurological disease, rheumatoid disease, anticoagulation medication), ASA-Score. Treatment related factors consisted of type of treatment, type of fracture according to Vancouver classification, time until surgery, duration of surgery, time of surgery (regular working hours 8 AM to 6 PM h vs. on call hours), intraoperative and postoperative complications and necessity of revision surgery.

Preoperative radiographs were classified by the surgeons according to the Vancouver classification. Type of treatment was categorized into conservative treatment, nail or plate osteosynthesis, replacement of prosthesis and more invasive treatment (proximal femoral replacement). Surgery-related criteria were only determined for patients with surgical treatment.

Mortality data was acquired from the German Residential Registry. Patients who emigrated during the observation period were excluded as they were no longer documented in the residential registry. Patient related factors as well as treatment related factors were assessed using the institutional database.

### Statistical analysis

Statistical Analysis was performed using SPSS for Windows (Version 29). Primary outcome was the all-course mortality rate 12 and 24 months after the event. First, a univariate statistical analysis was conducted using the Kaplan-Meier Estimator and log-rank test. A p-value < 0.050 was considered significant in univariate and multivariate analysis. Several criteria were identified that showed significant impact on overall survival. Those criteria were reviewed in a multivariate analysis using Cox-Regression. Only cases with information for all significant criteria were included in the multivariate model, i.e. excluding cases with conservative treatment. To assess the difference between treatment methods another multivariate model was built only containing criteria not related to surgical treatment.

## Results

As of the final mortality data retrieval on November 30, 2023 (mean: 48 months postoperatively, range: 0.23 to 152 months), 92 out of 158 patients (58.2%) had deceased. The one year mortality rate was 23.4%, and the two year mortality rate was 29.2%.

**Fig. 1:**
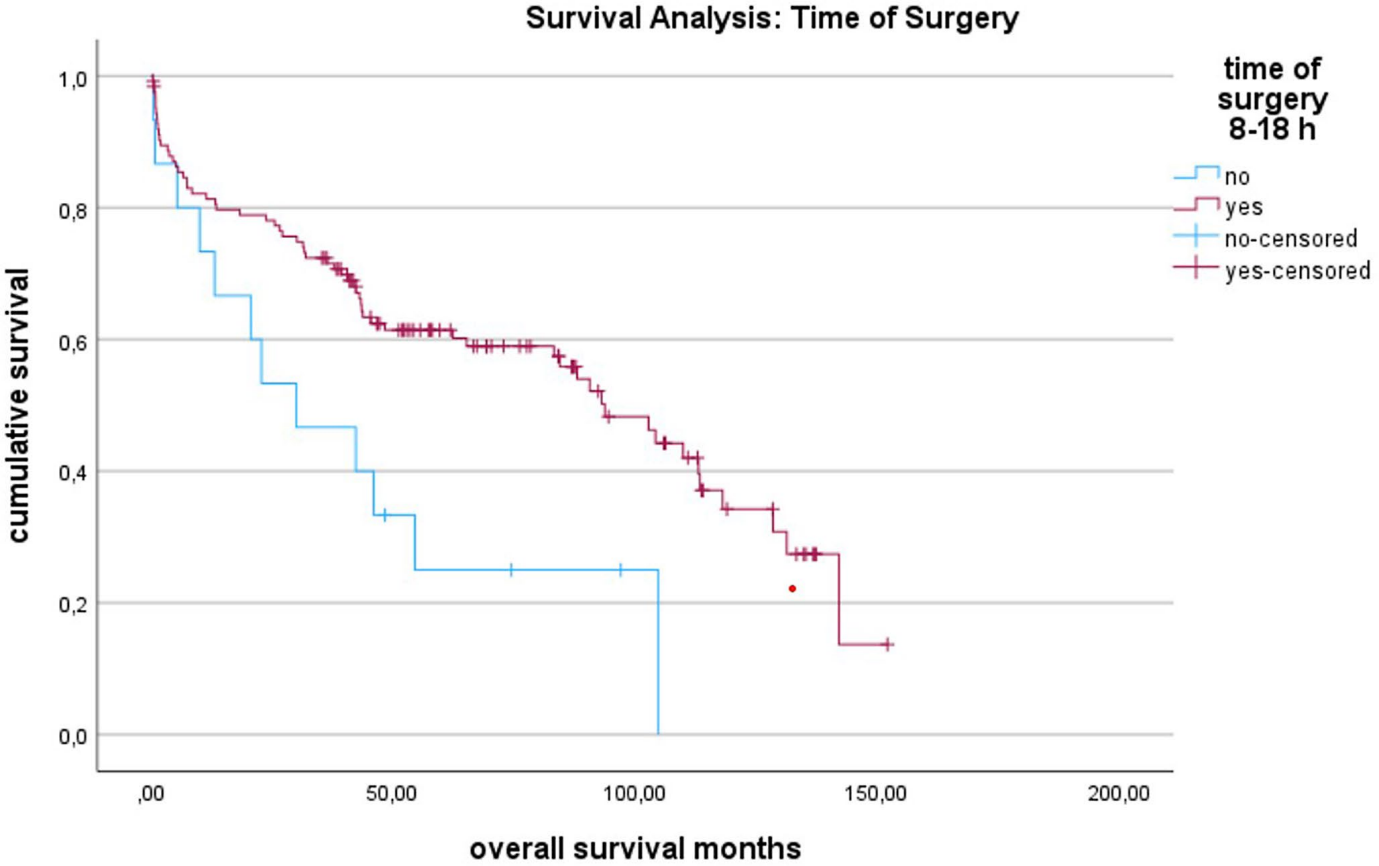
Kaplan Meier analysis for time of surgery

Multivariate statistical analysis revealed a 66.4% lower mortality for males (OR: 0.336, 95% CI 0.174, 0.646; *p* = 0.001). Mortality increased by 12.9% for each additional year of patient age (OR: 1.129, 95% CI 1.078, 1.182; *p* < 0.001). Additionally, the presence of cardiovascular disease increased mortality (OR: 3.39, 95% CI 1.020, 11.267; *p* = 0.046), as did dementia (OR: 4.98, 95% CI 2.551, 9.714; *p* < 0.001) and neurological diseases (OR: 1.996, 95% CI 0.994, 4.008; *p* = 0.052). Primary THA with a standing time of over 105 months was associated with a 1.993 times higher chance of mortality (OR: 1.993, 95% CI 1.110, 3.578; *p* = 0.023). A threshold of 105 months was chosen as this was the mean standing time of the primary THA in the cohort. Surgery performed during regular working hours (8:00 AM to 6:00 PM) was associated with a 53.2% lower chance of mortality compared to surgeries conducted during night duty hours (OR: 0.468, 95% CI 0.223, 0.968; *p* = 0.046; Fig. 1).

**Fig. 2:**
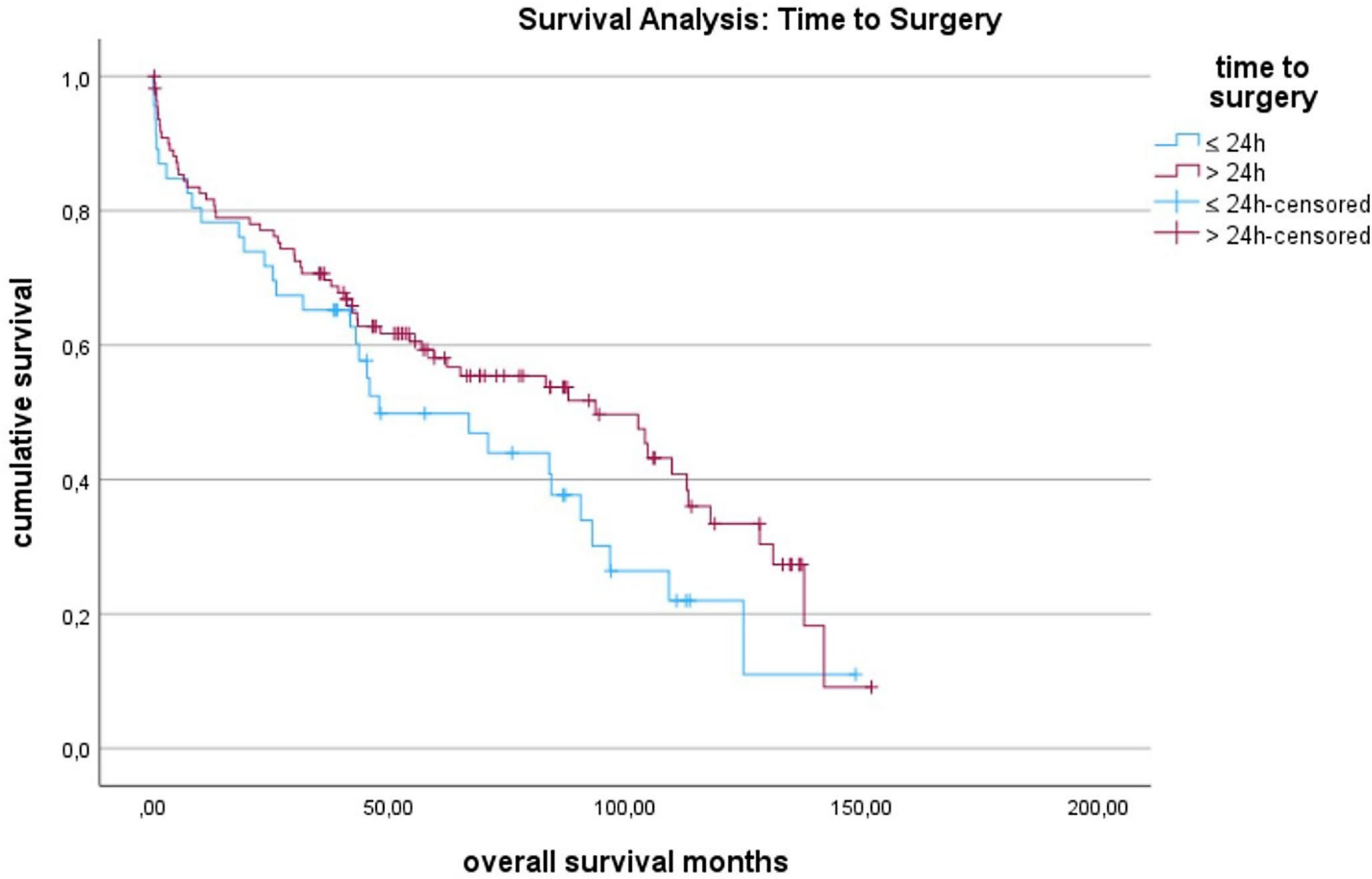
Kaplan Meier analysis for time to surgery

The treatment options used were as follows: conservative treatment 12/158 cases (7.6%), plate fixation in 73 cases (46.2%), revision arthroplasty in 55 cases (34.8%), proximal femoral replacement in 12 cases (7.6%), non-plate fixation four cases (2.5%) and cement spacer only in one case (0.6%). There were no intramedullary nails used. A secondary model was constructed to assess the treatment options recommended by the Vancouver classification. There was no significant difference in mortality between conservative treatment and plate fixation (*p* = 0.763). However, conservative treatment versus revision arthroplasty showed a 2.531 times higher mortality rate (*p* = 0.002), and plate fixation versus revision arthroplasty also showed a higher mortality rate (OR 2.812, *p* = 0.002). The type of fracture according to the Vancouver classification did not significantly impact mortality (*p* = 0.179). The mean time to surgery was two days, and this variable was not significant in either the univariate nor multivariate analysis (*p* = 0.541; Fig. 2), (Table. 1 and 2).

**Table 1:** Baseline characteristics

	Survived overall survival	Deceased overall survival	*p*	OR
N	66 (41.8%)	92 (58.2%)		
Age [per y]^1^	72.92 (SD ± 11.039 )	81.26 (SD ± 7.921)	**< 0.001**	1.129 (95% CI 1.078, 1.182)
Sex [f: m]^1^	43:23 (65%:35%)	59:33 (64%:36%)	**0.001**	0.336 (95% CI 0.174, 0.646)
BMI^2^	28.76 (SD ± 6.686)	25.81 (SD ± 4.693)	**0.003**	
THA standing time [m]^1^	98.82 (SD ± 96.897)	117.18 (SD ± 96.497)	**0.021**	1.993 (95% CI 1.110, 3.578)
Comorbidities				
Cardiovascular disease^1^	49 (74.24%)	87 (94.57%)	**0.046**	3.390 (95% CI 1.020, 11.267)
neurological disease other than dementia^1^	8 (12.12%)	19 (20.65%)	**0.052**	1.996 (95% CI 0.994, 4.008)
dementia^1^	3 (4.55%)	24 (26.09%)	**< 0.001**	4.978 (95% CI 2.551, 9.714)
pulmonary disease^3^	8 (12.12%)	16 (17.39%)	0.704	
rheumatoid disease^3^	1 (1.52%)	5 (5.43%)	0.487	
anticoagulation^2^	21 (31.82%)	53 (57.61%)	**< 0.001**	
ASA^2^	2.32 (SD ± 0.589)	2.81 (SD ± 0.631)	**0.001**	
Fracture type (Vancouver)^3^			0.179	
B1^3^	15 (22.73%)	21 (22.83%)		
B2^3^	31 (46.97%)	32 (34.78%)		
B3^3^	6 (9.09%)	6 (6.5%)		
C^3^	8 (12.12%)	19 (20.65%)		
Treatment^2^			**0.009**	
Nonoperative^1^	1 (1.52%)	11 (11.96%)		
Plate (ORIF)^1^	25 (37.88%)	48 (52.17%)		
Revision arthroplasty^1^	35 (53.03%)	20 (21.74%)		
Time to surgery^3^	5.4769 (SD ± 14.2567)	4.0625 (SD ± 5.26114)	0.541	
Surgery within working hours^1^	62 (95.38%)	63 (84%)	**0.046**	0.468 (95% CI 0.223, 0.968)

1 Multivariate analysis (p value from Cox-Regression)

2 Factor only significant in univariate analysis (p value from Kaplan Meier Estimator)

3 Factor not significant, p value from Kaplan Meier Estimator

**Table 2:** Odds of survival for different treatments

Treatment ^2^	*p*	OR
Nonoperative vs. Plate	0.763	1.111 (95% CI 0.560, 2.204)
Nonoperative vs. Revision arthroplasty	**0.002**	2.531 (95% CI 1.421, 4.507)
Plate vs. Revision arthroplasty	**0.007**	2.812 (95% CI 1.318, 5.998)

## Discussion

The aim of this study was to determine which patient and treatment-related factors influence mortality following periprosthetic fractures (PPF) around the hip.

In this cohort, the one year all-cause mortality rate (23.4%) as well as the two year all-cause mortality rate (29.2%) were higher than reported in the meta-analysis by Lamb et al. (13.4%) [12]. However, Khan et al. reported a one year-mortality for PPF of 21% [10]. The mortality observed in this cohort is similar to the one year-mortality in for femoral neck fractures [12, 21]. Therefore, PPF – like other hip fractures – must still be regarded as a fracture type associated with high mortality in geriatric orthopaedic surgery.

The patient and treatment related factors that remained significant in the multivariate analysis largely align with the identified risk profile for acquiring PPF and previous findings concerning mortality in PPF [8, 15]. This study did not find a significant impact of type of primary joint replacement on the overall survival compared to other studies [1, 9, 11]. While understanding the patient related risk profile is important, that most of the significant factors cannot be modified in the limited timeframe until surgery. Thus, these insights are valuable for preventing future PPF.

A frequently discussed topic is whether the time to surgery is as crucial in PPF as it is in other hip fractures [4, 6, 7, 14, 19, 20]. A recent meta-analysis suggested a negative impact of delayed surgery on 30-day mortality and complications [7]. Another study concluded that surgery beyond 48 h after admittance might result in more surgical and medical complications [14]. Conversely, it was also reported that delayed surgery for more than 48 h had no statistically significant impact on mortality and morbidity, allowing more time for careful planning [6, 19, 20]. In our study, the time to surgery also showed no significant impact on overall survival. Given the contradictory evidence, caution is needed when drawing conclusions about the ideal time to surgery in PPF.

**Fig. 3:**
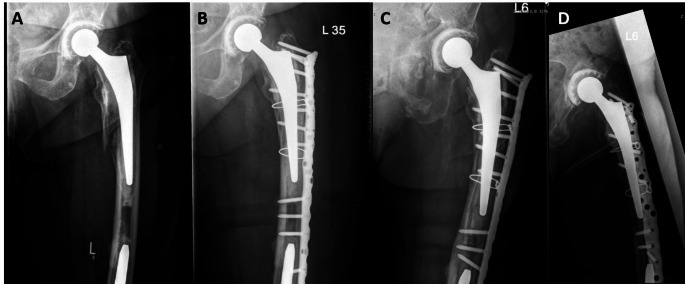
88 years-old female patient with a Vancouver B1 periprosthetic femur fracture who was operated during daytime. **A**) AP radiograph of the left hip after fall from standing height. **B**) AP radiograph at postoperative day 2 after locking plate fixation. **C**) + **D**) AP and axial radiograph 2 years postoperatively showing a healed fracture

**Fig. 4:**
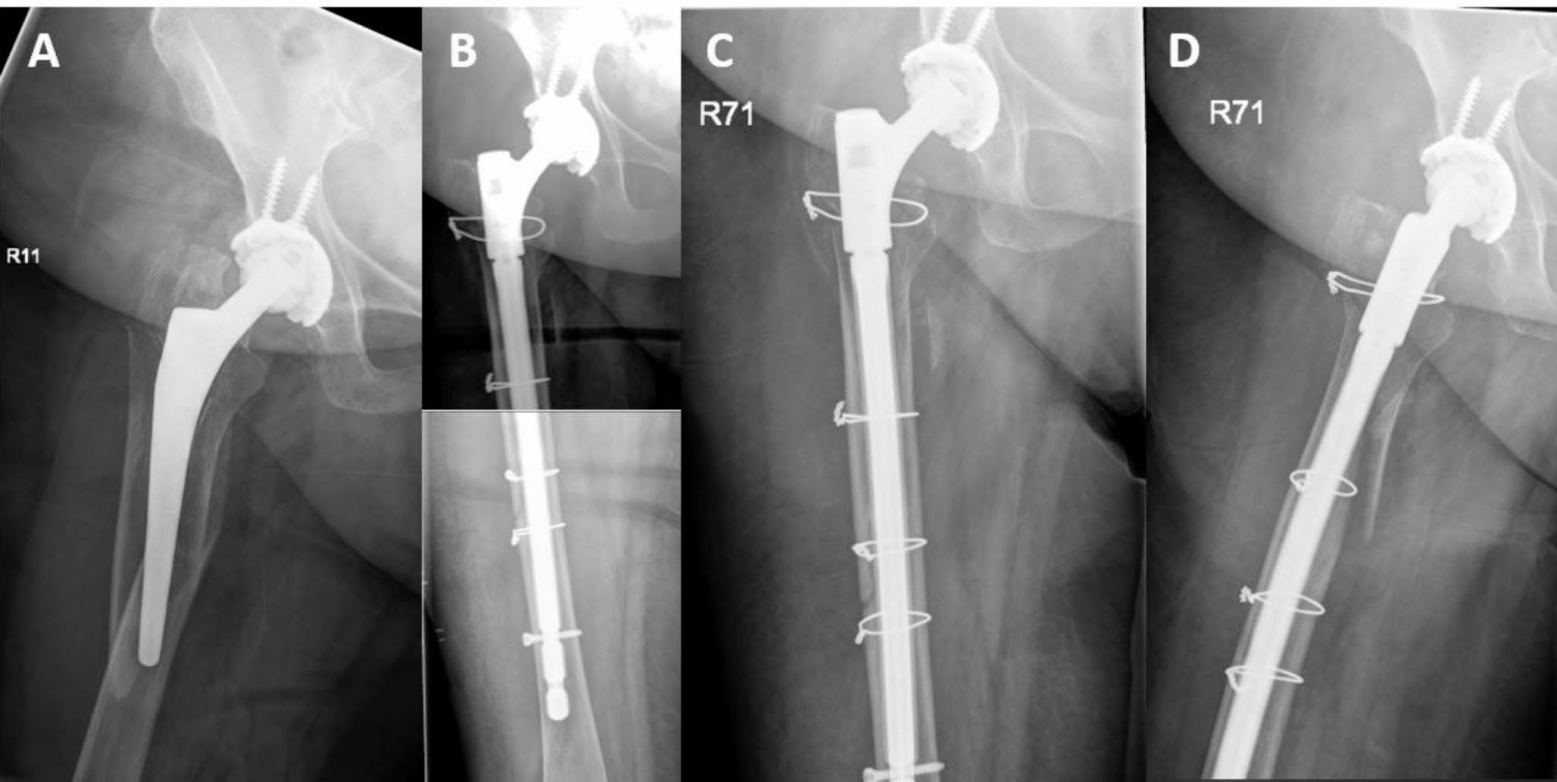
88 years-old female patient with a Vancouver B3 periprosthetic femur fracture who was operated during on-call hours. **A**) AP radiograph of the right hip after fall from standing height. **B**) AP radiograph at postoperative day 5 after revision arthroplasty. **C**) + **D**) AP and axial radiograph 6 weeks postoperatively showing stable conditions

Fewer studies have explored the importance of the time of surgery. Our results demonstrate a survival advantage for patients with surgical treatment during regular working hours (8 AM to 6 PM). This suggests that the treatment of PPF benefits from a better staffing and more preparation during these hours. These findings support the approach of considering PPF as early elective surgery rather than urgent surgery, implying that thorough planning is more critical for outcomes than a short time to surgery (see Figs. 3 and 4). However, to validate these conclusions, more - preferably prospective - studies need to be conducted.

Generally, the choice of treatment for PPF around the hip is based on the Vancouver classification and modified treatment algorithms [5, 22]. Our results show an advantage for PPF treated with revision arthroplasty versus plate fixation and conservative treatment. These findings should be interpreted with caution, as factors besides the Vancouver classification might influence the choice of treatment, such as anesthesiologic clearance or patient consent to surgery. These factors may hinder adherence to treatment algorithms and potentially distort the survival evaluation of different treatment options.

The limitations of this study consist of the retrospective and monocentric design. Patients in this study received treatment at a university hospital, where care was provided by a team consisting of two junior residents, one senior resident, and a board-certified consultant specializing in orthopaedic and trauma surgery, even during on-call hours. This level of staffing cannot, of course, be guaranteed in peripheral hospitals. Therefore, the results cannot be generalized to all hospitals across different levels of care. This argues against a uniform legal requirement for all hospitals. Such regulations should be tailored to the available resources and expertise. While all consultants have experience with the osteosynthesis of periprosthetic fractures (PPFs), surgeons on duty during on-call hours do not always have experience in revision arthroplasty. Additionally, not all revision implants are always readily available in the hospital.

Furthermore, no physical follow-up appointments or examinations were conducted; mortality data were obtained from the residential registry. However, due to the mandatory report of death to the residential registry, the follow-up of all-cause mortality was 100%. Postoperative complications may not have been recorded at our institution if patients were treated elsewhere.

Given the mortality rate in this study, comparable to other hip fractures, further research is necessary to optimize the treatment of PPF. Surgery during regular working hours could potentially improve mortality, but these findings need further investigation with larger cohorts and multi-center, prospective study design.

## Conclusion

Surgical treatment during regular working hours is associated with lower mortality compared to surgery outside these hours. In this retrospective cohort, time to surgery showed no significant impact on all-cause mortality, and revision arthroplasty was associated with lower mortality than conservative treatment or plate fixation.

## Data Availability

(data transparency): Data will not be made available.

## Code Availability

(software application or custom code): not applicable.
